# Effects of tacrolimus treatment on the gut microbiota and metabolites in liver transplant recipients

**DOI:** 10.1371/journal.pone.0343817

**Published:** 2026-02-26

**Authors:** Guohui Wang, Lu Liu, Hanshu Zhang, Panpan Mao, Saijuan Lu, Xiaofang Zhang, Xingde Li, Cangsang Song

**Affiliations:** Department of Pharmacy, The First People’s Hospital of Kunming City and Calmette Affiliated Hospital of Kunming Medical University, Kunming, Yunnan, China; The University of Texas Rio Grande Valley School of Medicine, UNITED STATES OF AMERICA

## Abstract

**Background:**

Liver transplantation (LT) is an effective treatment for patients with end-stage liver disease. In recent years, more and more evidence has supported the association between gut microbiota dysbiosis and the pathogenesis and progression of liver diseases.

**Methods:**

The study included 36 patients who received tacrolimus treatment after liver transplantation. Patients were stratified into subgroups according to three key variables: tacrolimus treatment duration, whole-blood tacrolimus concentration, and tacrolimus concentration-to-dose (C/D) ratio. Fecal samples and whole-blood specimens were collected from all participants. The Illumina HiSeq X platform was used to detect the gut metagenome, analyzing the composition and characteristics of the gut microbiota. Liquid chromatography-tandem mass spectrometry (LC-MS/MS) technology was employed to detect metabolites of the gut microbiota, revealing their metabolic profiles.

**Results:**

As the duration of tacrolimus use increased, the diversity of the gut microbiota also increased, and the abundance of *Escherichia coli_D* and *Bacteroides stercoris* rose. Additionally, the abundance of *Brunovirus* and *Uetakevirus* tended to decrease. The abundance of gene functions related to chemical carcinogenesis and bacterial invasion of epithelial cells significantly decreased. In the gut microbiota metabolites, 16 substances like Astragaloside A and Acetyl-L-carnitine significantly increased, while 108 substances like Capsaicin and TLK significantly decreased. Within a certain range, as the concentration of tacrolimus in whole blood increased, the diversity of the gut microbiota increased. The abundance of *Phocaeicola* and *Klebsiella* increased, and the abundance of *Peduovirus* among viruses also rose. However, excessively high concentrations may lead to a decrease in the diversity of the gut microbiota and a decrease in the abundance of *Phocaeicola*. With respect to the C/D ratio, increased ratios were linked to significantly higher levels of 57 fecal metabolites (e.g., PC 34:2, 5-Methyl-2’-deoxycytidine), whereas 13 metabolites (e.g., FAHFA 2:0/16:0) showed substantial declines.

**Conclusions:**

Tacrolimus treatment is associated with distinct alterations in gut microbiota and metabolites among LT recipients. These findings provide a preliminary framework for future investigations aimed at optimizing immunosuppressive regimens, although their clinical translational potential requires validation in larger-scale, prospective cohort studies.

## Introduction

Liver transplantation (LT) is an effective treatment for patients with end-stage liver disease, liver failure, and hepatocellular carcinoma (HCC), and it can significantly improve the long-term survival rate of these patients [1]. However, the survival rate of LT recipients is significantly affected by various complications, including rejection reactions and infections. In particular, opportunistic infections play a crucial role in influencing the prognosis of transplantation [2]. While virtually any bacterial species can cause infections in LT patients, the most prevalent pathogens by far are *Enterococcus species*, *Viridans streptococci*, *Staphylococcus aureus*, and various members of the *Enterobacteriaceae* family [3]. When the gut microbiota is imbalanced and the gut barrier function is compromised, bacteria within the gut could easily translocate, which may lead to corresponding infectious infectious complications [4].

The gut microbiota refers to the vast community of microorganisms residing in the animal gut. Its components or metabolites can interact with the liver through various gut-related mechanisms [5]. Anatomically, the liver can regulate intestinal function via the bile duct, while the gut can modulate liver function through the portal vein. Additionally, these two organs appear to indirectly influence each other's functions through the systemic circulation, establishing a bidirectional relationship among the liver, gut, and their microbiota, known as the “gut-liver axis” [6,7]. After LT, recipients are prone to gut microbiota dysbiosis, which is influenced by multiple factors. On the one hand, the transplantation surgery itself alters the physiological state of the gut-liver axis. For example, choledochojejunostomy is more likely to result in abnormal bile excretion than choledochocholedochostomy. This affects the nutritional supply and colonisation environment for the gut microbiota. On the other hand, postoperative medications might directly or indirectly disrupt the microbiota structure [8, 9]. Existing studies have observed dynamic changes in the composition of the gut microbiota in LT recipients during the perioperative period. On average, microbial diversity decreases during the first three weeks after transplantation, before gradually increasing during the observation period [10].

At present, preliminary studies have explored the association between post-transplant immunosuppressants and the gut microbiota. However, there are still obvious limitations, and the specific impact of tacrolimus on the gut microbiota and metabolites in LT patients remains unclear. From the perspective of research progress, existing achievements mainly focus on three aspects: First, the macroscopic impact of immunosuppressants on microbiota composition. Led by J Casper Swarte, the team used shotgun metagenomic sequencing to analyzed fecal samples from 415 LT recipients. They found that postoperative use of tacrolimus combined with mycophenolate mofetil (MMF) and antibiotics led to a decrease in gut microbiota diversity and an increase in the abundance of *Bacteroides* [11]. In a diabetic mouse model, tacrolimus was shown to induce an increase in the abundance of *Escherichia coli* and *Bacteroides* in the gut, while reducing the proportion of *Bifidobacterium*. This was accompanied by changes in fecal short-chain fatty acid metabolites [12]. Second, the potential association between gut microbiota and tacrolimus metabolism. A study in the field of kidney transplantation found that *Faecalibacterium prausnitzii* may directly metabolize tacrolimus into the less active metabolite M1. The abundance of this bacterium is positively correlated with the oral dose of tacrolimus [13]. This suggests that the gut microbiota may affect the efficacy and toxicity of tacrolimus through metabolic regulation. Third, the overall changes in gut microbiota function after LT. Through metabolomic analysis, Bajaj JS et al. found that the gut microbiota function of recipients was significantly improved after successful LT included reduce endotoxin synthesis, restoration of ammonia metabolism pathways, and recovery of the ability to regulate the enterohepatic circulation of bile acids [14].

Further research should explore the dynamic interaction between the metabolism of immunosuppressive drugs and the gut microbiota. This could enable personalised immunosuppressive therapy for transplant recipients. Such personalisation could reduce the risk of post-transplant infections and graft loss [15]. However, the shortcomings of existing studies are also quite evident, and these are precisely the gaps that this study aims to fill. The research design lacks dimensional diversity. Most studies only group subjects by “duration of medication use” and fail to incorporate clinical monitoring indicators related to tacrolimus, such as “dose - blood concentration - individual metabolism”. This makes it impossible to distinguish the differential effects of different levels of drug exposure on the microbiota. Meanwhile, the research techniques and analytical depth are limited. Most studies focus solely on the composition of the microbiota; while a small number of studies integrate metabolomics, they do not establish a linked analysis of the “microbiota - functional genes - metabolites”. Furthermore, none of these studies explore the association between viral communities and tacrolimus.

LT patients require long-term administration of immunosuppressants to continuously prolong the survival time of both the transplanted liver and the recipients [16]. Tacrolimus (TAC), a calcineurin inhibitor, serves as the core agent in current immunosuppressive regimens and is widely used as the first-line treatment for liver and kidney transplants. Its conventional administration protocol involves combination with mycophenolate mofetil (MMF) and glucocorticoids [17]. Tacrolimus has a narrow therapeutic window: insufficient dosage may trigger immune rejection, while excessive dosage may increase the risks of nephrotoxicity, neurotoxicity, and other adverse effects [18]. Therefore, precise regulation of its dosage is of great importance. The role of the gut microbiota in modulating drug efficacy provides a new perspective for optimizing tacrolimus administration regimens. Although there is currently no direct evidence confirming an association between gut microbiota dysbiosis and tacrolimus metabolism, indirect evidence supports this possibility [19]. For instance, increased bioavailability of oral tacrolimus after LT may lead to diarrhea [20]. Virus-induced intestinal inflammation may damage intestinal epithelial cells, thereby resulting in abnormally high tacrolimus concentrations in transplant patients’ blood [21].

The aim of this study is to investigate the impact of tacrolimus on the gut microbiota of LT patients. Patients were grouped based on the duration of tacrolimus use, whole blood drug concentration, and concentration-dose ratio. The Illumina HiSeq X platform was employed to detect the gut metagenome, analyzing the composition and characteristics of the gut microbiota. Liquid chromatography-tandem mass spectrometry (LC-MS/MS) technology was used to measure gut microbiota metabolites and reveal their metabolic profiles. The study aims to uncover the effects of tacrolimus on host gut microbial function and metabolites. While not directly guiding immediate clinical adjustments, these findings provide a scientific basis that may guide the long-term goal of optimizing immunosuppressive therapy for LT patients after longitude, multicenter, large sample size studies validate them in the future.

## Methods

### 1. Participants

From November 2023 to August 2024, the subjects of the study included 36 LT patients in hospital from the First People's Hospital of Kunming City, who were enrolled in this study after signing the informed consent form. All patients had received tacrolimus-based immunosuppressive therapy after LT. Gut fecal and whole blood samples were collected from LT patients receiving different doses of drug treatment and at different postoperative time points. The Ethics Committee of the First People's Hospital of Kunming City & Calmette Affiliated Hospital of Kunming Medical University (No. YLS2023−50), Written informed consent was obtained from all individual participants.

Inclusion criteria were defined as: (1) Patients who underwent orthotopic LT; (2) Received tacrolimus-based immunosuppressive therapy after transplantation; (3) Voluntarily participated in the study and signed the informed consent form. Exclusion criteria were: (1) Complicated with other organ transplantation; (2) Severe infections (e.g., sepsis, severe pneumonia) within 1 month before sample collection; (3) Received probiotic, prebiotic, or antibiotic treatment within 2 weeks before sample collection; (4) Complicated with severe gastrointestinal diseases (e.g., inflammatory bowel disease, intestinal obstruction); (5) Pregnancy or lactation; (6) Mental disorders or inability to cooperate with sample collection and clinical data documentation.

Fecal and whole blood samples were collected from all participants, who were stratified based on different postoperative time points and tacrolimus dosage regimens. Key clinical variables, including patient age, surgical technique (conventional vs. piggyback), type of biliary reconstruction, immunosuppressant regimens, and antibiotic usage, were documented.

### 2. Sample collection

Fecal samples were collected once from each participant at least 1 month after LT and when they were in a stable clinical state (no active infection or rejection): participants were instructed to urinate first to avoid contamination, defecate into a sterile container, immediately use the sterile spoon attached to the sampling tube to collect 3–5 g of the middle section of feces (to minimize air exposure), place the collected feces and the spoon together into a cryogenic tube containing 5 mL of fecal preservation solution, seal the tube tightly and label it with the participant's ID and collection time, temporarily store it at −20°C (for no more than 2 hours) before transferring it to −80°C for long-term storage, and ship the sample to the laboratory on dry ice within 1 week.

After the participant had fasted for 8–12 hours, 5 mL of antecubital venous blood was collected using a vacuum blood collection tube containing EDTA-K2. The tube was immediately gently inverted 5–8 times to mix the blood (avoiding vigorous shaking to prevent hemolysis). Within 30 minutes of collection, the mixed whole blood was aliquoted into pre-labeled cryogenic tubes using a sterile pipette (recommended aliquot volume: 500 μL to 1 mL per tube), pending tacrolimus whole blood concentration testing and subsequent analysis. Detailed records of the sample collection time, sample ID, processing procedures, and any abnormal conditions were maintained throughout the entire process.

### 3. Analytical methods

(1) Tacrolimus Blood Concentration Monitoring: Blood concentration monitoring of tacrolimus was performed in patients who had received tacrolimus for at least 3 days. In the early morning under fasting conditions, 2 mL of venous blood was collected and placed into EDTA-K2 vacuum tubes for subsequent analysis. Tacrolimus concentrations were measured using the i1000 analyzer with the corresponding reagent kits, strictly following the standard operating procedures of the i1000. Internal quality control was implemented throughout the assays.
(2) Metagenomic sequencing: Microbial DNA was extracted from fecal samples using the E.Z.N.A.® stool DNA Kit (Omega Bio-tek, Norcross, GA, U.S.), following the manufacturer's instructions. Metagenomic sequencing libraries were constructed and sequenced at Shanghai Biozeron Company. The process is briefly described as follows: 1 μg of DNA was fragmented into approximately 450 bp segments using a Covaris S220 ultrasonic machine (Woburn, MA USA) and then subjected to PE150 sequencing on the Illumina HiSeq X platform. Quality control was performed using Trimmomatic (https://github.com/usadellab/Trimmomatic) software to remove low-quality and contaminated reads. The reads that passed quality control were mapped to the human genome (version: hg19) using the BWA mem algorithm to remove host contamination and obtain clean reads [22]. Bacterial annotation was performed by aligning with the UHGG database [23], and viral annotation was done by aligning with the MGV database [24]. The BLASTN tool was used to compare with the UHGG database to identify and quantify the microbial composition.
(3) Collect intestinal microbiota samples and immediately place them into sterile EP tubes, with complete labeling of information and shortening the interval between collection and processing to maintain the integrity of the microbiota. Add 80% methanol aqueous solution to each EP tube, operate carefully to avoid splashing, and ensure thorough mixing. Subsequently, immerse the EP tubes in liquid nitrogen for quick-freezing for 3–5 minutes to terminate reactions and maintain the integrity of analytes; after removal, thaw them at room temperature or in a water bath with a temperature ≤ 37°C. Once thawed, vigorously vortex for 1–2 minutes to disrupt cell membranes, and then use an ultrasonic cleaner or probe-type ultrasonic disruptor for ultrasonic treatment for 5–10 minutes to enhance extraction. Transfer the treated samples to centrifuge tubes, centrifuge at 10,000–15,000 rpm at 4°C for 10–15 minutes, and carefully collect the supernatant containing the target analytes. Transfer the supernatant to a suitable container, and freeze-dry it at 0.01–0.05 mbar and −50 to −80°C for 8–12 hours until it becomes a dry powder. Add 100–200 μL of methanol to the dry residue and vortex to dissolve [24]. Finally, load the samples into the Vanquish UHPLC system (Thermo Fisher, Germany) coupled with the Orbitrap Q Exactive™ HF mass spectrometer (Thermo Fisher, Germany) at Biozeron Co., Ltd. (Shanghai, China), set the chromatographic and mass spectrometric parameters according to the characteristics of the target analytes, and perform three or more parallel analyses to ensure the reproducibility of the results [25].
(4) Metabolite Identification and Quantification: The raw data files were processed using the CD3.1 library search software, where each metabolite was subjected to a preliminary screening based on parameters such as retention time and mass-to-charge ratio. Subsequently, relevant information was set for peak extraction, and metabolite identification was performed by comparing the high-resolution tandem mass spectra with databases including mzCloud, mzVault, and MassList. The identified metabolites were statistically categorized according to the ClassyFire (http://classyfire.wishartlab.com/) classification information. Functional and categorical annotations of the identified metabolites were conducted using databases such as the Kyoto Encyclopedia of Genes and Genomes (KEGG, https://www.genome.jp/kegg/pathway.html), the Human Metabolome Database (HMDB, https://hmdb.ca/metabolites), and LIPID MAPS (https://www.lipidmaps.org/). Chromatographic peak integration was carried out for the detected peaks in the samples, with background ions removed using blank samples. The raw quantitative results were normalized using the total peak area. Metabolites with a Coefficient of Variation (CV) of less than 30% in the Quality Control (QC) samples were retained for the final identification and relative quantification results of the metabolites.

### 4. Statistical analysis

(1) Diversity Analysis: The abundance and diversity of the microbial community can be reflected by single-sample diversity analysis (Alpha diversity). The mothur indices (version v.1.30.1; http://www.mothur.org/wiki/Schloss_SOP#Alpha_diversity) were used to calculate the Shannon, ACE, Chao1, and Simpson indices. The Chao index is used to estimate species richness, the Shannon index and Simpson index calculate species diversity, and the ACE index is an index that estimates species diversity based on rare species; a higher value indicates a greater variety of species in the community. Using R language (https://www.r-project.org/) to plot a boxplot that displays the differences in diversity indices under different treatments. The differences between the two groups were assessed using the Wilcoxon rank-sum test (P ≤ 0.05). The variation among the three groups was evaluated using the Tukey’s HSD test (using the “multcomp” package, P ≤ 0.05). Statistical analysis was performed on the abundance of bacteria and viruses at different taxonomic levels (phylum, class, order, family, genus, and species). The degree of difference in sample species abundance distribution can be quantified through statistical distances. The Bray-Curtis algorithm was used to calculate the distance between sample pairs, resulting in a distance matrix. QIIME (https://qiime.org/) was used to compute the beta diversity distance matrix. R language was employed for principal co-ordinates analysis (PCoA) statistical analysis and plotting to study the similarity of sample community composition. Non-parametric multivariate statistical tests (Adonis) were performed using R vegan package to assess the statistically significant differences of bacterial community diversity indices between samples. Differences were considered significant at p < 0.05.
(2) Variation Analysis: Before the analysis, since many species were detected only in individual samples, especially viruses, to reduce false positives, species with a detection rate of less than 60% in the study samples were first removed. Subsequently, the Wilcox rank-sum test (p value ≤ 0.05), random forest, and LEfSE (https://huttenhower.sph.harvard.edu/lefse/) analysis were used to identify potential key species that could distinguish between groups at various levels of bacteria and viruses, respectively, to screen for usable species for subsequent analyses. In the functional gene analysis, the Wilcox rank-sum test and random forest analysis were employed to identify differentially expressed functional genes. To ensure data quality and comparability, QC samples were used to monitor system stability. Signal drift was corrected using QC-based LOESS normalisation, followed by total ion current (TIC) normalisation. Features exhibiting poor reproducibility in QC samples (RSD > 30%) were excluded from subsequent analyses. Based on the metabolite abundance results of each sample, the metabolic profiles of positive and negative ion identifications for grouped samples were combined, and Orthogonal Partial Least Squares Discriminant Analysis (OPLS-DA) was used to filter out orthogonal signals that were not related to model classification. The permutation test was performed on OPLS-DA (MetaboAnalyst, https://github.com/xia-lab/MetaboAnalystR). We applied univariate analysis (t-test) to calculate the statistical significance (P-value). The metabolites with P-value< 0.05 and fold change ≥2 or FC ≤ 0.5 were considered to be differential metabolites. Volcano plots were used to filter metabolites of interest which based on log2 (Fold Change) and -log10 (p-value) of metabolites by ggplot2 in R language. The lollipop chart screens were used to filter metabolites of interest which based on P-value values tested by VIP and T-test. Association statistical analysis and figures were performed using the Biozeron Cloud Platform [26].
(3) Functional Gene Study: Based on the annotation results from the UHGG and MGV databases, which correspond to Clusters of Orthologous Groups of proteins (COG) and the Kyoto Encyclopedia of Genes and Genomes (KEGG), proteins were functionally classified according to COG and KEGG annotations to obtain the functional abundance of COG and KEGG. According to sample grouping, potential functional genes that could distinguish between groups were identified at the COG and KEGG L1, L2, and L3 levels, and the viable functional genes were selected for subsequent analysis.

## Results

### 1. Associations between tacrolimus exposure and gut microbiota composition in liver transplant recipients

**Fig 1:**
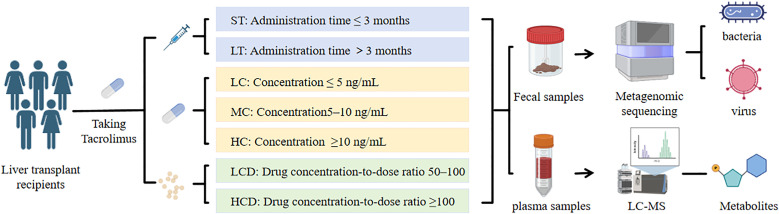
Experimental design for stratification of tacrolimus-treated liver transplant patients and multi-omics sample analyses. Fig 1 illustrates the experimental design where liver transplant patients taking tacrolimus were divided into ST and LT groups based on medication duration, and into LC, MC, and HC groups (N = 3) based on the drug concentration of tacrolimus in whole blood, as well as into LCD and HCD groups based on the drug concentration-to-dose ratio. Fecal samples were collected for metagenomic testing, and whole blood samples were analyzed for metabolomics.

To explore the associations between tacrolimus exposure and gut microbiota of LT recipients, we collected fecal samples from 36 LT patients who received tacrolimus-based immunosuppressive therapy postoperatively. The clinical characteristics of these patients are summarized in **Table 1**. Patients were grouped based on the duration of tacrolimus use: the short-term use group (ST group, n = 21, ≤ 3 months) and the long-term use group (LT group, n = 15, > 3 months). As the core immunosuppressant in LT therapy, tacrolimus has a narrow therapeutic window. Direct measurement of whole blood tacrolimus concentration can reflect the actual exposure level of the drug in the systemic circulation of recipients at the time of sampling. According to the tacrolimus concentration in whole blood samples, the patients were further divided into three groups: the low concentration group (LC group, n = 19, whole blood tacrolimus concentration ≤5 ng/ml), the medium concentration group (MC group, n = 14, whole blood tacrolimus concentration 5–10 ng/ml), and the high concentration group (HC group, n = 3, whole blood tacrolimus concentration ≥10 ng/ml). A key limitation of analyzing concentration alone is the inter-individual variability in drug metabolism. By grouping patients based on the concentration-dose ratio, the impact of individual metabolic capacity on the gut microbiota can be isolated. Individuals were grouped based on the drug concentration-to-dose ratio (drug dose per kilogram of body weight, C/D): those with a C/D ratio of 50–100 were classified into the low C/D ratio group (LCD group, n = 7), and those with a C/D ratio >100 were classified into the high C/D ratio group (HCD group, n = 17). The remaining samples were excluded due to the lack of patient weight data or a C/D ratio <50 (**Fig 1**). The cases with a concentration-dose ratio <50 are mostly attributed to abnormally high individual metabolic efficiency in patients, which may be accompanied by other confounding factors. These factors could independently affect the gut microbiota and metabolites, rather than being driven solely by the C/D ratio.

**Table 1:** Clinical characteristics of liver transplant recipients.

Summary of Clinical Characteristics of Liver Transplant Recipients		
Characteristic		Data (n = 36)
Age (years)		Range: 5–71, Median: 54
Sex, n (%)	Male	33 (91.7%)
	Female	3 (8.3%)
Post-Transplantation Time	≤ 3 months	21(58.3%)
	> 3 months	15(41.7%)
Biliary Reconstruction Type	Duct-to-duct anastomosis:	24 (66.7%)
	Not specified	12 (33.3%)
Tacrolimus Dosing	Total Daily Dose (mg)	Range: 0.5–7.0; Median: 4.0
	Daily Dose per Kilogram (mg/kg)	Range:0.01 ~ 0.15 mg/kg; Median: 0.055
Concomitant Immunosuppressant	Mycophenolate Mofetil/Sodium	36 (100%)
Surgical Technique	Conventional	36 (100%)
Portal Hypertension, n (%)	Yes	15 (41.7%)
	No	9 (25.0%)
	Not specified	12 (33.3%)

Note: Data were collected from 36 liver transplant recipients who received tacrolimus-based immunosuppressive therapy between November 2023 and August 2024 at the First People's Hospital of Kunming City.

**Fig 2:**
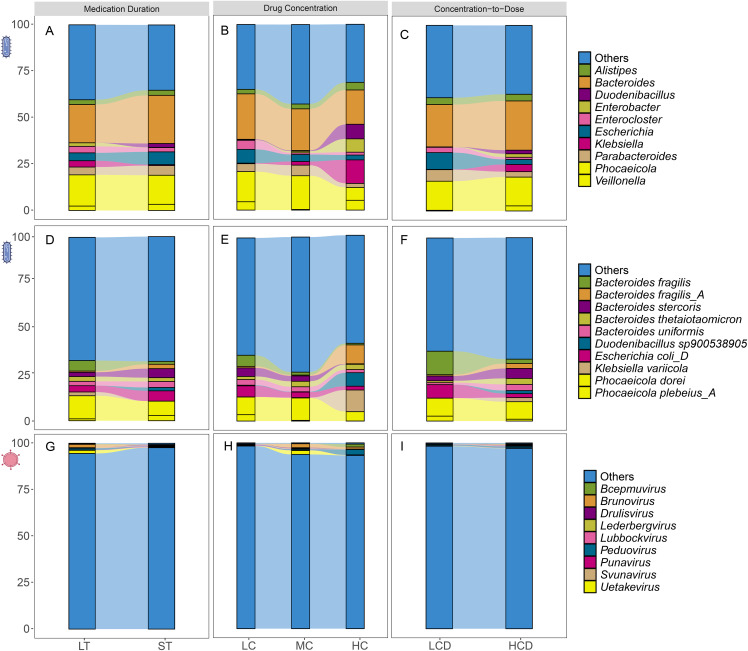
Relative abundances of dominant bacteria and viruses in tacrolimus-treated LT patient subgroups. Panel A, Panel D, Panel G show the bar graphs of the relative abundance of dominant bacteria at the genus and species levels and dominant viruses at the genus level for the ST and LT groups. Panel B, Panel E, Panel H show the bar graphs of the relative abundance of dominant bacteria at the genus and species levels and dominant viruses at the genus level for the LC, MC, and HC (N = 3) groups. Panel C, Panel F, Panel I show the bar graphs of the relative abundance of dominant bacteria at the genus and species levels and dominant viruses at the genus level for the LCD and HCD groups.

The overall composition of the gut microbiota in the study cohort was first characterized. At the bacterial genus level, the top 10 most abundant taxa in post-treatment LT patients were *Bacteroides, Phocaeicola, Escherichia, Parabacteroides, Enterocloster, Veillonella, Alistipes, Klebsiella_A, Klebsiella,* and *Prevotella* (**Fig 2A**). At the species level, the top 10 abundant bacterial species in LT patients after medication were *Phocaeicola dorei, Escherichia coli_D, Bacteroides fragilis, Bacteroides stercoris, Bacteroides uniformis, Bacteroides thetaiotaomicron, Klebsiella_A michiganensis, Phocaeicola plebeius_A, Bacteroides xylanisolvens,* and *Bacteroides ovatus* (**Fig 2D**). At the viral genus level, the top 10 abundant viral genus in LT patients after medication were *Brunovirus, Uetakevirus, Peduovirus, Lubbockvirus, Punavirus, Drulisvirus, Lederbergvirus, Svunavirus, Bcepmuvirus,* and *Teseptimavirus* (**Fig 2G**).

Intergroup comparisons suggested a trend toward associations between tacrolimus treatment duration and gut microbial composition. Relative to the ST group (≤3 months of tacrolimus use), the LT group (>3 months of tacrolimus use) exhibited increased abundance of *Bacteroides* and *Escherichia* and decreased abundance of *Phocaeicola* at the bacterial genus level (**Fig 2A**). At the species level, the abundance of *Escherichia coli_D* and *Bacteroides stercoris* increased, while the abundance of *Phocaeicola dorei* and *Bacteroides fragilis*decreased (**Fig 2D**). At the viral genus level, the abundance of *Brunovirus* and *Uetakevirus* decreased (**Fig 2G**). Comparisons based on different whole blood concentrations of tacrolimus found that at the bacterial genus level, with increasing whole blood drug concentrations, the abundance of *Bacteroides* and *Escherichia* gradually decreased, while the abundance of *Klebsiella* increased (**Fig 2B**). At the species level, *Klebsiella varicola* and *Bacteroides fragilis_A* had increased abundance in the HC (N = 3) group and were negligible in the MC group and LC group, with *Phocaeicola dorei* being most abundant in the MC group (**Fig 2E**). At the viral genus level, the abundance of *Peduovirus* increased in the HC (N = 3) group, while *Brunovirus* and *Uetakevirus* were relatively more abundant in the MC group (**Fig 2H**).

In the C/D ratio stratification, higher C/D ratios were significantly associated with alterations in gut microbial composition. At the bacterial genus level, the abundance of *Bacteroides* increased with increasing C/D ratio, while the abundance of *Escherichia* and *Parabacteroides* decreased (**Fig 2C**). At the species level, the abundance of *Bacteroides fragilis* and *Escherichia coli D* decreased in the HCD group (**Fig 2F**). At the viral genus level, the abundance of *Peduovirus* increased in the HCD group (**Fig 2I**).

### 2. Associations between tacrolimus exposure and gut microbial diversity

**Fig 3:**
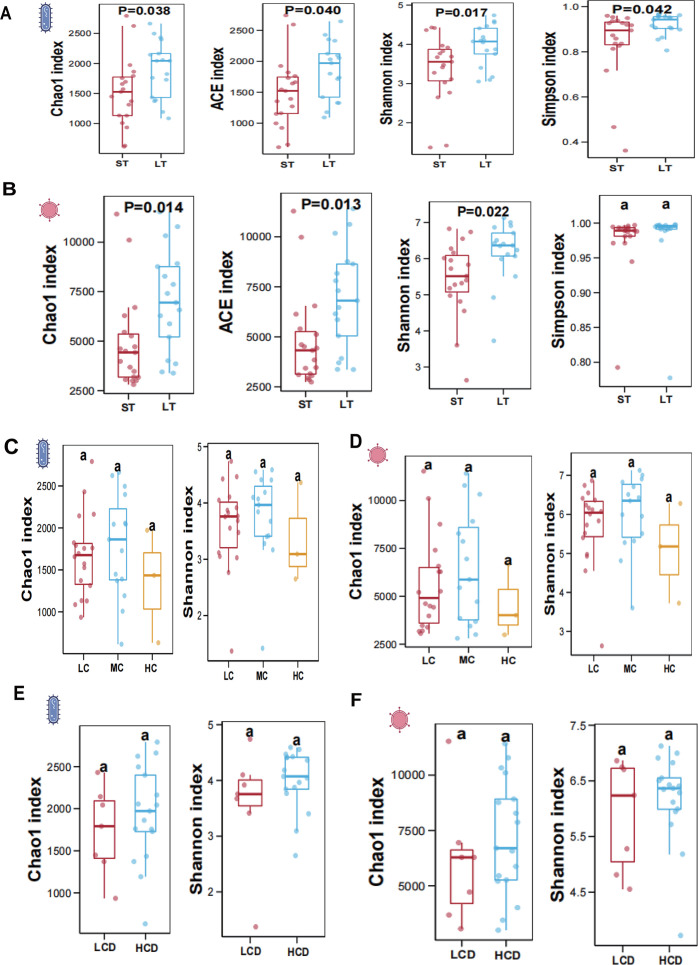
Gut bacterial and viral α-diversity comparisons across tacrolimus-treated liver transplant patient subgroups. Panel A presents a comparison of the gut bacterial α-diversity indices between the ST and LT groups across four different indices: the Chao1 index, ACE index, Shannon diversity index, and Simpson index. Each subplot features box plots that show the distribution range, median, and quartiles of the data for each group, with p-values less than 0.05 on each box indicating significant differences between groups. Panel B shows the comparison of gut viral α-diversity indices between the ST and LT groups across the same four indices. Panel C-F respectively display the Chao1 index and Shannon diversity index for bacteria and viruses in the LC, MC, and HC (N  =  3) groups, as well as the LCD and HCD groups. Note: The “a” labels in the figure are formatting artifacts and can be ignored.

To compare the richness and evenness of gut microbiota among the groups, four different indices (Chao1 index, ACE index, Shannon index, and Simpson index) were used to compare across different groups. The comparison results showed that the species level of bacteria, the index values of the LT group were all higher than those of the ST group, with Chao1 index (p = 0.038), ACE index (p = 0.040), Shannon index (p = 0.017), and Simpson index (p = 0.042) (**Fig 3A**). At the species level of viruses, the index values of the LT group were all higher than those of the ST group. There were significant differences between the ST and LT groups for the Chao1 index (p = 0.014), ACE index (p = 0.013), and Shannon index (p = 0.022). However, for the Simpson index (p = 0.764), indicating no statistically significant difference (**Fig 3B**).

In contrast, no significant differences in bacterial and viral diversity among the different tacrolimus blood concentration groups across the indices (all p > 0.05) (Fig 3C-3D). Similarly, no significant differences in bacterial or viral diversity were found between the LCD and HCD groups (all p > 0.05) (Fig 3E-3F).

**Fig 4:**
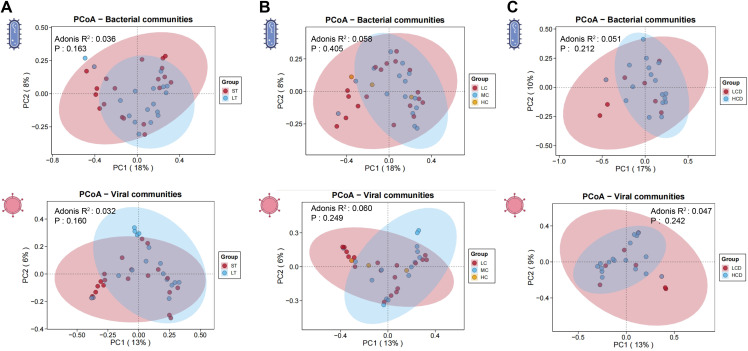
PCoA Plots and PERMANOVA (Adonis) analysis of gut microbiota dissimilarities in stratified patient cohorts. Panel A-C present the PCoA analysis for the different groups, reflecting the differences in the data on a two-dimensional coordinate plot, with the axes representing the two principal components that maximize the variance. Samples with more similar compositions are closer together on the PCoA plot. The results of the PERMANOVA (Adonis) analysis (R² and p-values) are shown in the figure.

To further explore the diversity differences of gut microbiota among the groups, we performed PCoA analysis. In the drug administration time groups, the ST and LT groups showed almost complete overlap in bacterial communities, with high similarity between the two groups. For viral communities, there was a high degree of overlap, indicating similar community structures (**Fig 4A)**. In the different blood drug concentration subgroups, the bacterial communities in the gut mostly overlapped, with high similarity across all groups. The viral communities showed large overlap in the middle part, with relatively high similarity, but the distribution trends varied, and there were some differences with changes in concentration (**Fig 4B)**. In the different C/D ratio subgroups, we found that the bacterial communities in the LCD and HCD groups were almost identical, and the viral communities completely overlapped (**Fig 4C)**.

### 3. Associations Between tacrolimus exposure and discriminatory gut microbiota taxa, and microbial functional gene profiles

**Fig 5:**
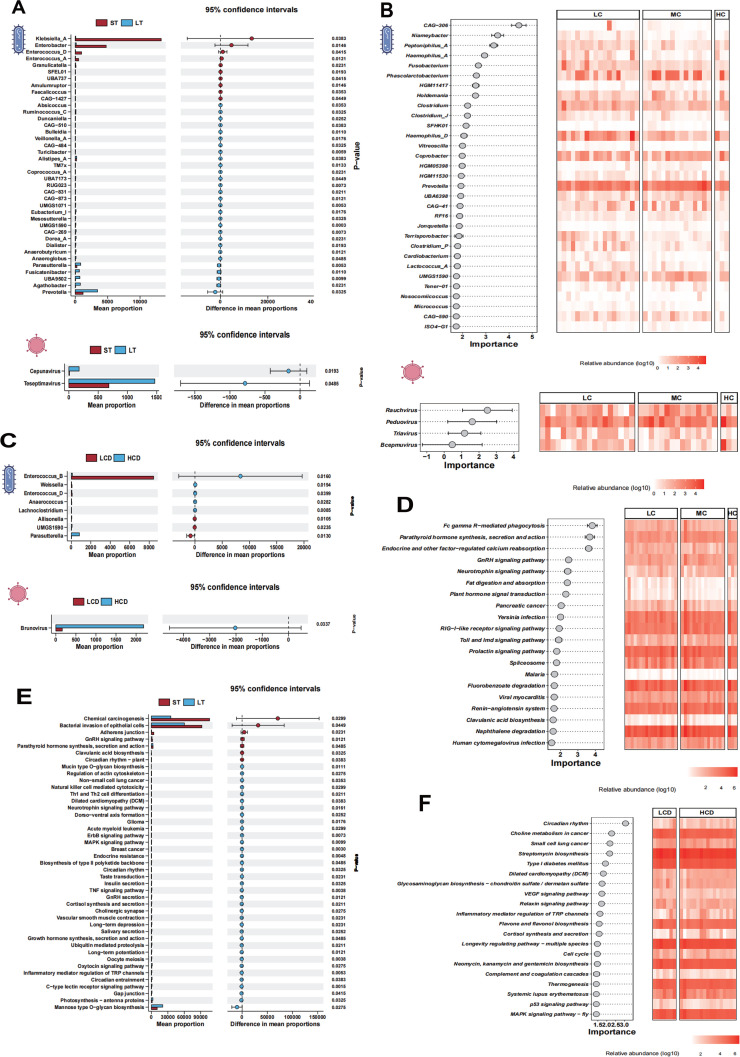
Identification and analysis of key biomarkers, functional traits, and their abundance changes across patient subgroups. Panel A, C are combination plots of biomarker abundance changes between groups as determined by the wilcox rank-sum test. The left bar graphs show the average abundance of identified key species in the study groups, with the X-axis representing the average proportion and the Y-axis listing different bacterial genera. Red squares represent Group ST, and blue squares represent Group LT. The right scatter plots show the abundance changes of key species between groups, with the X-axis displaying the difference in average proportions. The numeric value on the far right is the P-value obtained from the Wilcox rank-sum test between species groups. Panel B shows the key species in different blood drug concentration groups, with the left dot plot displaying the identified Biomarkers and their importance to the grouping, retaining results with importance higher than 1.5. The right heatmap uses a color gradient to show the abundance of Biomarkers in the study samples, with redder colors indicating higher abundance, and gray representing the absence of the Biomarker in the sample. Panel D, F list multiple features on the left, related to the duration of medication. Each feature has different red shaded areas in each group, indicating the relative importance of the feature in these two classifiers. The darker the color, the higher the importance of the feature in the classifier. Panel E is a combination plot of key functional abundance changes between groups as determined by the Wilcox rank-sum test. The left chart shows the average proportion for each process, with red representing Group ST and blue representing Group LT. The right chart shows the average difference and 95% confidence intervals for these processes between the two groups.

Based on the sample grouping, potential key discriminatory taxa that distinguish intergroup differences were identified. In the medication duration-based grouping, the most significant differential bacteria at the genus level were *Klebsiella_A* and *Enterobacter*, with high abundance in the ST group. The significant differential viruses at the genus level were *Cepunavirus* and *Teseptimavirus*, with higher abundance in the LT group (**Fig 5A**). For the grouping by different blood drug concentrations, the most significant differential bacteria *CAG-306* and the significant differential virus *Rauchvirus* were of the highest importance in the respective groups (**Fig 5B**). In the grouping by different C/D ratios, the primary differential bacteria at the genus level were *Enterococcus_B* with high abundance in the LCD group, and the main significant differential virus was *Brunovirus* with the highest abundance in the HCD group (**Fig 5C**).

To characterize the functional features of the gut microbiota, a non-redundant gene set from metagenomic sequencing data, gene functional classification and annotation were performed, and inter-group differences were analyzed. In the blood drug concentration groups, gene functions such as Fc gamma R-mediated phagocytosis, Parathyroid hormone synthesis, secretion and action, and Endocrine and other factor-regulated calcium reabsorption were of higher importance (**Fig 5D**). In the tacrolimus treatment duration subgroups, the chemical carcinogenesis and bacterial invasion of epithelial cells were significantly higher in the ST group than in the LT group, and the Mannose type O-glycan biosynthesis gene function was significant lower in the ST group compared with the LT group (**Fig 5E**). In the C/D ratio subgroups, the circadian rhythm gene function was of high importance (**Fig 5F**).

### 4. Liver transplantation recipients treated with tacrolimus exhibited differences in gut microbial metabolites

**Fig 6:**
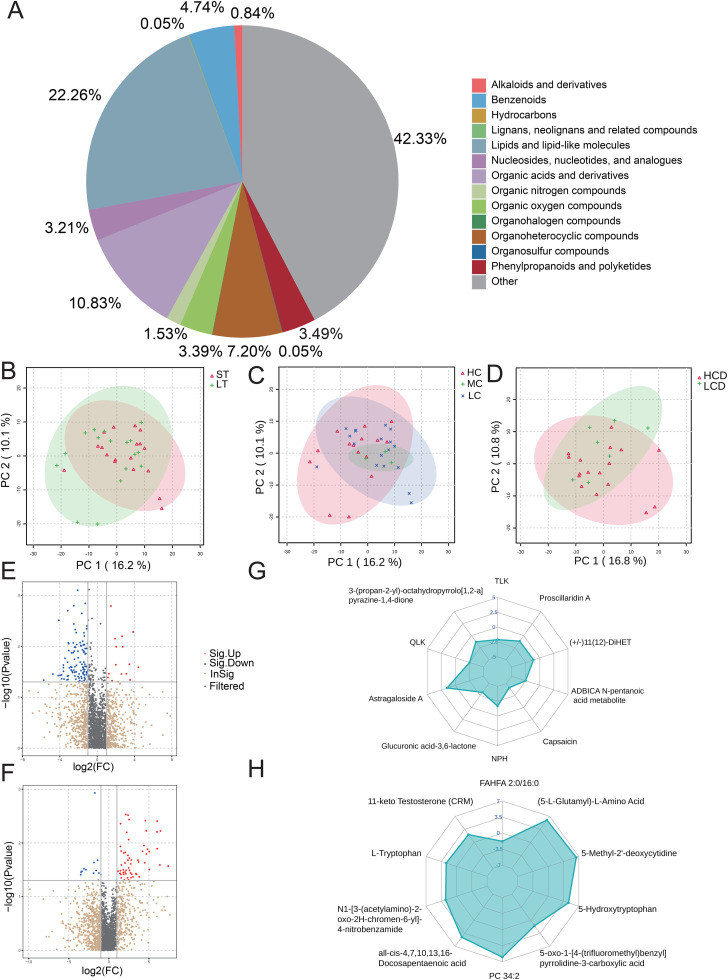
Profiling of metabolite categories and differential metabolite analysis (PCA, Volcano Plots, Radar Plots) in tacrolimus-treated cohorts. Panel A shows each color representing a metabolite classification, with unknown classifications represented as Other. The percentage indicates the proportion of metabolites belonging to this type out of all identified metabolites. Panel B-D are PCA score plots. After dimensionality reduction analysis, samples have relative coordinate points on the principal components PC1 and PC2. The distance between the coordinate points represents the degree of clustering and dispersion among samples; a closer distance indicates higher similarity between samples, while a greater distance indicates greater differences. PCA analysis can be used to observe the trend of separation between groups in the experimental model, as well as the presence of any outliers, and reflects the variability between and within groups from the original data. The confidence ellipses represent the “true” samples of the group, which are distributed within this area at a 95% confidence level; samples outside this area can be considered as potential outliers. Panel E, Panel F are volcano plots of differential metabolites for different medication duration and different drug concentration-to-dose ratio groups, respectively. These volcano plots use consistent screening thresholds: fold-change cutoff of |log₂(FC)| ≥ 1 (actual fold change ≥ 2 or ≤ 0.5) to distinguish biologically significant differences from experimental fluctuations, and independent samples t-test p < 0.05 to control the false positive rate. OPLS-DA model VIP ≥ 1 is additionally used as an auxiliary criterion to enhance screening accuracy. The color of the points in the graph represents the category of the metabolites, with red indicating significantly up-regulated metabolites that meet the threshold, blue indicating significantly down-regulated metabolites that meet the threshold, gray indicating metabolites that do not meet the fold change screening threshold, and tan indicating metabolites that meet the fold change threshold but do not meet the P-value threshold. Panel F, Panel G are radar plots of differential metabolites for different medication duration and different drug concentration-to-dose ratio groups, respectively. For each group comparison, we calculate the corresponding ratio for the quantitative values of differential metabolites and apply a logarithmic transformation with base 2, selecting the top 10 differential metabolites with significant changes (up and down regulation combined) for display. The grid lines correspond to the log_2_FC. The green colored area is formed by the lines connecting the dots (metabolites).

To explore the relationship between tacrolimus exposure and gut microbial metabolite patterns, metabolic profiles were compared across subgroups stratified by tacrolimus treatment duration, whole-blood concentration, and C/D ratio. All identified metabolites from the samples were classified and statistically analyzed based on the Classy Fire classification information. The analysis revealed that the most abundant class was Lipids and lipid-like molecules, accounting for 22.26%, followed by Organic acids and derivatives at 10.38% (**Fig 6A**). PCA analysis of the combined metabolite abundance results from each group showed that the groups with different medication durations basically overlapped, indicating similar metabolite profile between groups (**Fig 6B**). In the grouping by blood drug concentration, the HC group completely overlapped with the LC and MC groups, while the LC and MC groups mostly overlapped but showed different trends (**Fig 6C**). The groups divided by C/D ratio mostly overlapped, but the trend directions of the two groups were different (**Fig 6D**).

Subsequent screening and visualization of differential metabolites revealed distinct metabolite alterations associated with tacrolimus exposure variables. In the medication duration groups, with the extension of medication time, 16 metabolites including Astragaloside A significantly increased, while 108 metabolites including TLK, QLK, and Capsaicin significantly decreased (Fig 6E-6F); in the C/D ratio groups, with the increase of the drug C/D ratio, 57 metabolites including 11-keto Testosterone (CRM) and L-Tryptophan significantly increased, while 13 metabolites including FAHFA 2:0/16:0 significantly decreased (Fig 6G-6H).

### 5. Correlation analysis of the gut microbiota with faecal metabolites and clinical variables

**Fig 7:**
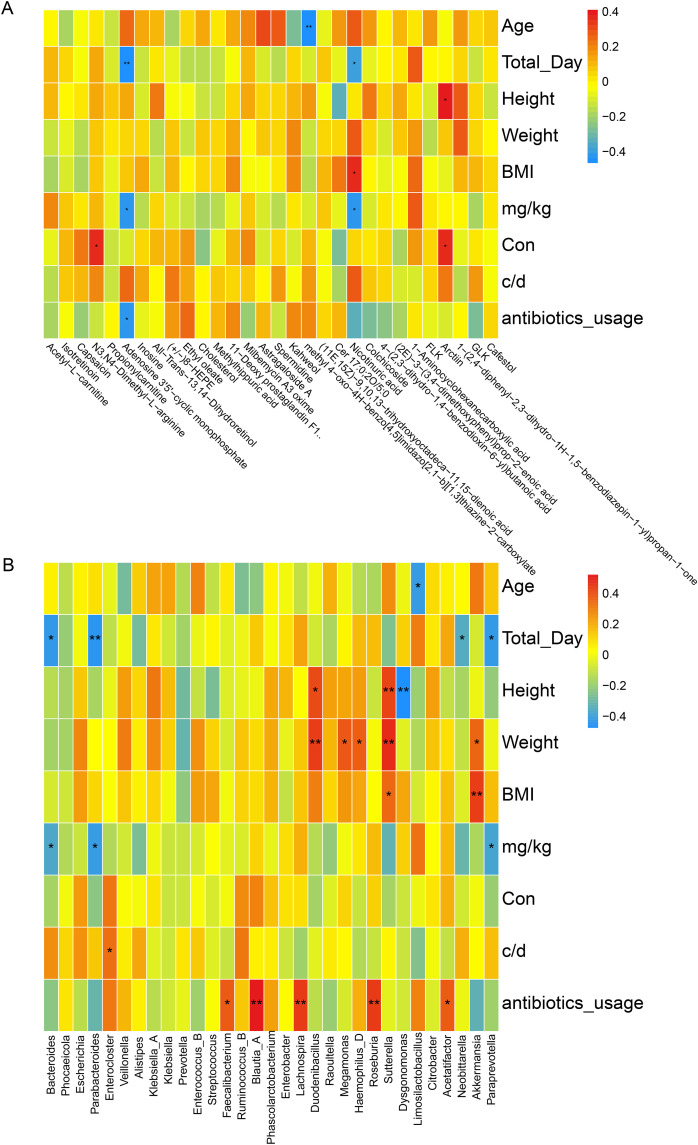
Spearman correlation analysis of clinical parameters, top 30 bacterial genera, and key differential metabolites. Panel A and Panel B show heatmaps of clinical indicators and the top 30 abundant genera, alongside the Spearman correlation coefficient for top 30 differentially accumulated metabolites (ST vs LT). The color scale shows the correlation coefficients, with red representing positive correlations and blue representing negative ones. Asterisks indicate statistical significance (* P < 0.05, ** P < 0.01).

To evaluate the associations between clinical variables on the gut microbiota and metabolome, we conducted a correlation analysis. Various clinical variables were found to be correlated with top 30 differentially accumulated metabolites (ST vs LT) to varying degrees (**Fig 7A**). Some metabolites showed significant correlations with BMI, height and dosing-related parameters (mg/kg, Con) (P < 0.05 or P < 0.01), whereas the correlations between age, antibiotic use and most metabolites were weaker or did not reach statistical significance. Regarding the correlations between the relative abundances of the top 30 bacterial genera (**Fig 7B**): Several bacterial genera showed significant correlations (P < 0.05 or P < 0.01) with multiple physical parameters (height, weight and BMI), whilst total day and antibiotic usage also exhibited significant positive or negative correlations with the abundance of certain genera. In contrast, age demonstrated weaker correlations or failed to reach statistical significance with most bacterial genera.

**Fig 8:**
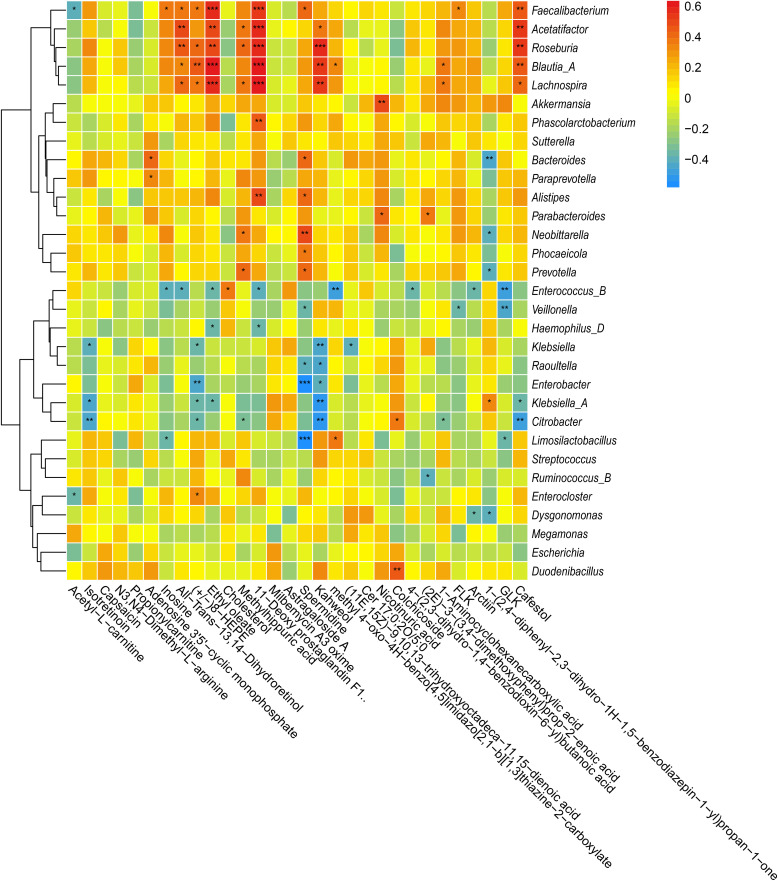
Heatmaps showing Spearman correlations between top 30 abundant genera and top 30 differentially accumulated metabolites. Heatmaps illustrate Spearman correlations between the top 30 abundant genera and the top 30 differentially accumulated metabolites (ST vs. LT groups). Red and blue colors on the scale represent positive and negative correlations, respectively, and asterisks indicate statistical significance (* P < 0.05, ** P < 0.01, *** P < 0.001).

Spearman’s rank correlation analysis was used to evaluate the associations between the predominant bacterial genera and the top 30 differentially accumulated metabolites (ST vs LT), and hierarchical clustering was performed based on the abundance of the genera. Distinct correlation patterns emerged between different genera and metabolites (**Fig 8**). Certain bacterial genera (e.g., Faecalibacterium, Roseburia, Blautia and Lachnospira) showed significant positive correlations with multiple metabolites, including cafestol and ethyl oleate. In contrast, several genera (e.g., Klebsiella, Enterobacter and Escherichia) predominantly displayed significant negative correlations with specific metabolites (P < 0.05 or P < 0.01) such as spermidine. Hierarchical clustering revealed that genera exhibiting similar correlation patterns clustered together.

## Discussion

The critical role of tacrolimus in immunosuppressive therapy following LT has been widely recognized, but its associations with the intestinal microecology of LT recipients remain unclear [27]. Given the importance of the gut microbiota in maintaining host health, this study aims to thoroughly analyze the effects of tacrolimus treatment on the gut microbiota and metabolism profiles in LT patients.

Our findings reveal that tacrolimus exposure is associated with alterations in gut microbial abundance and diversity among LT recipients. Specifically, prolonged tacrolimus treatment duration correlates with increased microbial diversity, accompanied by elevated abundances of *Escherichia coli* and *Bacteroides* and reduced abundances of *Phocaeicola dorei, Brunovirus, and Uetakevirus.* This pattern of increased diversity may reflect the gradual recovery of the gut microbiota following surgical trauma and illness, as previously observed in post-transplant cohorts [28]*.*

Notably, *Phocaeicola dorei* is a common member of the human gut microbiota with potential anti-inflammatory properties [29]. *Brunovirus* is not a formally defined independent virus genus by the ICTV, but rather represents an operational taxonomic unit (OTU/vOTU) within the bacteriophage group [30]. *Uetakevirus* members were reported as candidates for phage therapy against multidrug-resistant clinical *Salmonella serovars* [31]. In experimental mouse models, it has been observed that, following antibiotic disruption, *K. michiganensis* helps to maintain resistance to colonisation by pathogens such as *E. coli* in the gut. This is achieved through nutritional competition mechanisms that prevent pathogens from proliferating unchecked, thereby prolonging host survival [32]. *Peduoviruses* are temperate phages that infect members of the γ-proteobacteria, such as *E. coli* may modulate bacterial community dynamics [33].

Meanwhile, tacrolimus treatment correlates with reduced expression of genes involved in chemical carcinogenesis and decreased levels of inflammatory metabolites in the gut microbiota. A potential pattern identified in our data is that within a certain tacrolimus concentration range, microbial diversity shows a positive correlation with the abundance of specific taxa (e.g., *Phocaeicola*, *Klebsiella*), whereas a negative correlation emerges at extremely high concentrations. This suggests a potential dose-response relationship between tacrolimus concentration and gut microbiota, though this remains a preliminary observation requiring further investigation into underlying mechanisms and associations. This observation implies a potential non-linear dose-response relationship between tacrolimus concentration and gut microbiota profiles, although this finding remains preliminary and necessitates further investigation into the underlying mechanisms and causal associations.

The sample size of LT patients is limited, but these samples are particularly valuable due to the challenges of obtaining them and their precious clinical context. Despite the limited sample size, we employed advanced metagenomic sequencing techniques to conduct in-depth the genomic data of the microbiota, maximizing the information extracted from each individual sample. This approach not only effectively addresses the limitations posed by the small sample size but also highlights the innovative design of the study and the depth of data analysis. Furthermore, the uniqueness of LT patients and the difficulty of acquiring such samples enhance scientific value and clinical significance of our findings, underscoring the importanceof this research.

Metagenomic sequencing revealed that the use of tacrolimus affects the abundance of the gut microbiota in LT patients. In the gut microbiota of patients using tacrolimus after LT, *Bacteroides* accounted for the highest proportion, followed by *Phocaeicola* and *Escherichia*. After LT, the levels of gut bacteria such as *Bacteroides fragilis* and *Bacteroides thetaiotaomicron*, which drive Treg cell induction and differentiation, increase [34]. Kim and others have demonstrated gut microbiota dysbiosis in SLE mice treated with tacrolimus, such as increased relative abundance of *Alloprevotella*, *Clostridium*, *Enterorhabdus*, and *Escherichia* [35]. In the context of organ transplantation, tacrolimus induced gut microbiota dysbiosis in skin-grafted rats, showing a significant increase in the relative abundance of *Allobaculum*, *Bacteroides*, and *Lactobacillus* [36]. These research findings are consistent with our results, and we further explore the impact of tacrolimus drug concentration and duration of treatment on the gut microbiota of LT patients.

As the duration of tacrolimus use extends, the diversity of the gut microbiota in LT patients increases, with a rise in the abundance of *Escherichia coli_D* and *Bacteroides stercoris*. It is reported that *Escherichia coli_D* is associated with human enteric disease [37]. A previous clinical study investigated kidney transplants treated with tacrolimus, and under a median treatment time of 6 years, tacrolimus was found to cause gut dysbiosis, with a significant increase in the relative abundance of *Escherichia coli* [38]. However, as the blood concentration of tacrolimus increases, the abundance of *Bacteroides* and *Escherichia* genera in LT patients gradually decreases. The use of tacrolimus promotes the growth of *Faecalibacterium* and *Bifidobacterium*, while reducing the population of *Bacteroides-Prevotella* and *Enterobacteriaceae* [39]. Importantly, *Bacteroides-Prevotella* and *Enterobacteriaceae* are the main Gram-negative bacteria that produce lipopolysaccharide (LPS). The potential reduction in endotoxin levels reveals an additional role of immunosuppressants in altering microbial structure, which may affect the occurrence of complications after LT. Jennings and colleagues studied tacrolimus-induced gut microbiota dysbiosis in 24 patients within 3 months after heart transplantation, comparing the effects of high-dose (0.1 mg/kg/d and higher) versus low-dose (0.1 mg/kg/d and lower) tacrolimus. High-dose tacrolimus produced beneficial effects, characterized by higher levels of alpha diversity, and a significant increase in the relative abundance of *Akkermansia* and *Subdoligranulum.* This led to a reduction in inflammation, such as a decrease in serum TNF α levels [40]. Due to different patterns of gut microbiota dysbiosis, different doses of tacrolimus exert dose-dependent effects on the immune system. Higher doses may promote the increase of beneficial bacteria and Treg cells, and improve transplant outcomes, especially *Lactobacillus* which regulates Treg cell differentiation [41]. Conversely, lower doses may reduce antimicrobial peptides such as Reg3β, thereby increasing the risk of Gram-negative bacterial infections [42]. This is consistent with our results, where the abundance of Gram-negative bacteria such as *Bacteroides* and *Escherichia* genera decreased in the higher tacrolimus dose group. The study found that in the low, medium, and high tacrolimus dose groups, the medium-dose group saw an increase in Parabacteroides abundance, which reduced the abundance of *Escherichia* genus. Additionally, our study found that in the low, medium, and high tacrolimus dose groups exhibited increased *Parabacteroides* abundance, which was associated with reduced *Escherichia* genus levels. Jiang et al. studied the effects of low, medium, and high doses of tacrolimus on LT rats. They discovered that a medium dose of tacrolimus reduced the abundance of pathogenic bacteria (*Bacteroides-Prevotella* and *Enterobacteriaceae*) and increased the abundance of probiotics (*Faecalibacterium* and *Bifidobacterium*) in the gut [39]. Both *Faecalibacterium* and *Bifidobacterium* are bacteria that participate in immune regulation through the gut-liver axis feedback loop [43], suggesting medium-dose tacrolimus may regulate the balance of the gut microbiota, which helps to modulate the immune response.

Tacrolimus administration after LT may affect the fecal metabolome. After LT surgery, the number of *Bifidobacterium* genus decreases, while the numbers of *Bacteroides* and *Prevotella* increase, as well as the reduction in the numbers of *Firmicutes, Clostridia*, and *Bifidobacterium* [44]. Our study found that with the prolonged use of tacrolimus, the functional genes related to chemical carcinogenesis decrease. Compared to the general population, LT recipients have a 2–3 times higher risk of developing de-novo malignant tumors [45]. The gut microbial dysbiosis caused by LT leads to this risk of malignant tumors. This is because the gut microbiome may affect systemic inflammation and immune homeostasis, thereby increasing the host’s susceptibility to malignant tumors [46]. These observations highlight the potential relevance of tacrolimus-associated microbial alterations to long-term post-transplant outcomes. Considering the potential for cancer caused by immunosuppression, clinicians should consider therapeutic adjustment of the microbiome as a strategy to prevent or mitigate rejection and other complications after LT [47]. For instance, by regulating the gut microbiome, it is possible to prevent the suppression of anti-tumor immune responses due to persistent stimulation by gut antigens, thereby preventing the development of hepatocellular carcinoma (HCC) [48]. This therapeutic adjustment of the microbiome maybe achieved through probiotic supplementation or fecal microbiota transplantation (FMT). Although current research evidence is limited, the existing literature on the use of probiotics in LT recipients suggests that probiotics have shown some effectiveness in reducing the incidence of infections after LT [49]. Furthermore, FMT has shown benefits in the treatment of alcohol-related liver disease and hepatic encephalopathy, but its application in the field of LT has not yet been reported in research [50]. Despite these uncertainties, the association between specific microbiota and favorable outcomes in LT suggests that FMT and probiotics may have potential utility. As a cross-sectional study, our work cannot establish clinical utility, but the identified associations highlight potential targets for future mechanistic and interventional research (e.g., FMT) to inform personalized treatment paradigms.

The associations between clinical variables and the gut microbiota and metabolome suggest that these potential confounding factors are mildly associated. We believe that they did not systematically confound the core tacrolimus association patterns of interest. Regarding the associations between predominant bacterial genera and metabolites differentially expressed LT vs ST groups, the immune-related metabolite spermidine exhibited negative correlations with *Klebsiella*, *Enterobacter* and *Escherichia*. This suggests that expansion of these Enterobacteriaceae may contribute to regulating and depleting host polyamine metabolism. It is worth noting that dysregulated polyamine metabolism has been shown to be closely associated with the onset and progression of colitis and colorectal cancer [51]. Positive correlations have been found between metabolites such as cafestol and ethyl oleate, and butyrate-producing genera such as *Faecalibacterium*, *Roseburia*, *Blautia* and *Lachnospira*. This suggests that these metabolites may be linked to a gut environment that is metabolically favourable and enriched in commensal bacteria well-documented anti-inflammatory and host-beneficial functions [52]. These potential mechanistic connections require validation in larger-scale prospective studies.

In this study, although we have achieved certain research results, there are still some shortcomings. First, our study is cross-sectional. It shows associations, not causation. We cannot prove tacrolimus alone caused the changes. Other factors could also affect the microbiome. These include the type of biliary reconstruction, concomitant use of mycophenolate mofetil (MMF), and perioperative antibiotic administration-all of which are known to profoundly influence gut microbiota composition. In our cohort, 34 out of 36 patients used MMF, which precludes meaningful adjustment or subgroup analysis. Antibiotics were administered to 22 out of 36 patients. Our analysis could not fully separate these effects. Future studies should be longitudinal. Sampling should start pre-transplant to definitively distinguish which alterations in the gut microbiota and metabolome are specifically attributable to tacrolimus immunosuppression, as opposed to the collective impact of transplant surgery, peri-operative antibiotics, hospitalisation and the post-transplant clinical environment. Second, the sample size is small, which may limit the generalizability and reliability of the experimental results, as well as the statistical power to detect more subtle associations. The observed effect sizes and identified microbial and metabolite signatures require validation in larger, multi-center prospective cohorts. In the future, we plan to expand the sample size and conduct multicenter clinical studies. Third, the involved signaling pathways are not clear, and this experiment did not construct the corresponding mouse models for validation, nor did it delve into the mechanism for verification. This makes it difficult for us to gain a deep understanding of the mechanism by which tacrolimus affects the gut microbiota and its metabolites. Subsequent studies will involve animal experiments or molecular experiments to further investigate the intrinsic mechanisms. In future research, we will expand the sample size, elucidate the related pathways, and construct appropriate animal models to provide a more robust theoretical basis for clinical treatment.

## Conclusions

This study employed integrated metagenomic and metabolomic approaches to explore the associations between tacrolimus exposure and alterations in gut microbiota composition, diversity, functional gene profiles, and metabolite patterns among LT. The results showed that tacrolimus exposure (duration, blood concentration, C/D ratio) correlated with distinct changes in gut microbial composition, diversity, functional genes, and metabolites. These observational associations provide a scientific basis for developing microbiota-informed personalized immunosuppressive strategies. Due to the cross-sectional design, causal inferences are not possible, and clinical utility requires validation in large-scale prospective cohorts. Future research should elucidate the underlying mechanisms and the prognostic value of microbial/metabolite signatures to optimize individualized therapy for LT patients.

## Supporting information

S1 File Raw data. (XLSX)

## References

[1] Samstein B, Smith AR, Freise CE, Zimmerman MA, Baker T, Olthoff KM, et al. Complications and their resolution in recipients of deceased and living donor liver transplants: findings from the A2ALL cohort study. Am J Transplant. 2016;16(2):594–602. doi: 10.1111/ajt.13479 26461803 PMC4733444

[2] Pantea R, Bednarsch J, Schmitz S, Meister P, Heise D, Ulmer F, et al. The assessment of impaired liver function and prognosis in hepatocellular carcinoma. Expert Rev Gastroenterol Hepatol. 2024;18(12):779–94. doi: 10.1080/17474124.2024.2442573 39688572

[3] Shbaklo N, Tandoi F, Lupia T, Corcione S, Romagnoli R, De Rosa FG. Bacterial and viral infections in liver transplantation: new insights from clinical and surgical perspectives. Biomedicines. 2022;10(7):1561. doi: 10.3390/biomedicines10071561 35884867 PMC9313066

[4] Wirth U, Jiang T, Schardey J, Kratz K, Li M, Schirren M, et al. The role of microbiota in liver transplantation and liver transplantation-related biliary complications. Int J Mol Sci. 2023;24(5):4841. doi: 10.3390/ijms24054841 36902269 PMC10003075

[5] Li C, Cai C, Wang C, Chen X, Zhang B, Huang Z. Gut microbiota-mediated gut-liver axis: a breakthrough point for understanding and treating liver cancer. Clin Mol Hepatol. 2025;31(2):350–81. doi: 10.3350/cmh.2024.0857 39659059 PMC12016628

[6] Wang J, Wang X, Zhuo E, Chen B, Chan S. Gut‑liver axis in liver disease: From basic science to clinical treatment (Review). Mol Med Rep. 2025;31(1):10. doi: 10.3892/mmr.2024.13375 39450549 PMC11541166

[7] Giannelli V, Di Gregorio V, Iebba V, Giusto M, Schippa S, Merli M, et al. Microbiota and the gut-liver axis: bacterial translocation, inflammation and infection in cirrhosis. World J Gastroenterol. 2014;20(45):16795–810. doi: 10.3748/wjg.v20.i45.16795 25492994 PMC4258550

[8] Ponziani FR, Valenza V, Nure E, Bianco G, Marrone G, Grieco A, Pompili M, Gasbarrini A, Agnes S, Sganga G. Effect of liver transplantation on intestinal permeability and correlation with infection episodes. PLoS One. 2020 Jun 26;15(6): e0235359. doi: 10.1371/journal.pone.0235359 PMC7319319 32589654

[9] Schurich A, Berg M, Stabenow D, Böttcher J, Kern M, Schild H-J, et al. Dynamic regulation of CD8 T cell tolerance induction by liver sinusoidal endothelial cells. J Immunol. 2010;184(8):4107–14. doi: 10.4049/jimmunol.0902580 20212092

[10] Kato K, Nagao M, Miyamoto K, Oka K, Takahashi M, Yamamoto M, et al. Longitudinal analysis of the intestinal microbiota in liver transplantation. Transplant Direct. 2017;3(4):e144. doi: 10.1097/TXD.0000000000000661 28405600 PMC5381737

[11] Swarte JC, Li Y, Hu S, Björk JR, Gacesa R, Vich Vila A, et al. Gut microbiome dysbiosis is associated with increased mortality after solid organ transplantation. Sci Transl Med. 2022;14(660):eabn7566. doi: 10.1126/scitranslmed.abn7566 36044594

[12] Qian M, Jiang Z, Xu C, Wang L, Hu N. Changes in the gut microbiota and derived fecal metabolites may play a role in tacrolimus-induced diabetes in mice. Future Microbiol. 2025;20(3):237–46. doi: 10.1080/17460913.2024.2444761 39711145 PMC11812427

[13] Guo Y, Crnkovic CM, Won K-J, Yang X, Lee JR, Orjala J, et al. Commensal gut bacteria convert the immunosuppressant tacrolimus to less potent metabolites. Drug Metab Dispos. 2019;47(3):194–202. doi: 10.1124/dmd.118.084772 30598508 PMC6367689

[14] Bajaj JS, Kakiyama G, Cox IJ, Nittono H, Takei H, White M, et al. Alterations in gut microbial function following liver transplant. Liver Transpl. 2018;24(6):752–61. doi: 10.1002/lt.25046 29500907 PMC5992060

[15] Yan S, Yin X-M. Gut microbiome in liver pathophysiology and cholestatic liver disease. Liver Res. 2021;5(3):151–63. doi: 10.1016/j.livres.2021.08.001 35355516 PMC8963136

[16] Ortiz V, Loeuillard E. Rethinking immune check point inhibitors use in liver transplantation: implications and resistance. Cell Mol Gastroenterol Hepatol. 2025;19(1):101407. doi: 10.1016/j.jcmgh.2024.101407 39326581 PMC11609388

[17] Lentine KL, Smith JM, Hart A, Miller J, Skeans MA, Larkin L, et al. OPTN/SRTR 2020 annual data report: kidney. Am J Transplant. 2022;22 Suppl 2:21–136. doi: 10.1111/ajt.16982 35266618

[18] Holt CD. Overview of immunosuppressive therapy in solid organ transplantation. Anesthesiol Clin. 2017;35(3):365–80. doi: 10.1016/j.anclin.2017.04.001 28784214

[19] Zimmermann M, Zimmermann-Kogadeeva M, Wegmann R, Goodman AL. Mapping human microbiome drug metabolism by gut bacteria and their genes. Nature. 2019;570(7762):462–7. doi: 10.1038/s41586-019-1291-3 31158845 PMC6597290

[20] Lemahieu W, Maes B, Verbeke K, Rutgeerts P, Geboes K, Vanrenterghem Y. Cytochrome P450 3A4 and P-glycoprotein activity and assimilation of tacrolimus in transplant patients with persistent diarrhea. Am J Transplant. 2005;5(6):1383–91. doi: 10.1111/j.1600-6143.2005.00844.x 15888045

[21] Nakamura A, Amada N, Haga I, Tokodai K, Kashiwadate T. Effects of elevated tacrolimus trough levels in association with infectious enteritis on graft function in renal transplant recipients. Transplant Proc. 2014;46(2):592–4. doi: 10.1016/j.transproceed.2013.11.040 24656020

[22] Bolger AM, Lohse M, Usadel B. Trimmomatic: a flexible trimmer for Illumina sequence data. Bioinformatics. 2014;30(15):2114–20. doi: 10.1093/bioinformatics/btu170 24695404 PMC4103590

[23] Almeida A, Nayfach S, Boland M, Strozzi F, Beracochea M, Shi ZJ, et al. A unified catalog of 204,938 reference genomes from the human gut microbiome. Nat Biotechnol. 2021;39(1):105–14. doi: 10.1038/s41587-020-0603-3 32690973 PMC7801254

[24] Nayfach S, Páez-Espino D, Call L, Low SJ, Sberro H, Ivanova NN, et al. Metagenomic compendium of 189,680 DNA viruses from the human gut microbiome. Nat Microbiol. 2021;6(7):960–70. doi: 10.1038/s41564-021-00928-6 34168315 PMC8241571

[25] Yuan M, Breitkopf SB, Yang X, Asara JM. A positive/negative ion-switching, targeted mass spectrometry-based metabolomics platform for bodily fluids, cells, and fresh and fixed tissue. Nat Protoc. 2012;7(5):872–81. doi: 10.1038/nprot.2012.024 22498707 PMC3685491

[26] Wang N, Li Y, Han S, Zhang Y, Yang J, Yin Z, et al. CFViSA: A comprehensive and free platform for visualization and statistics in omics-data. Comput Biol Med. 2024;171:108206. doi: 10.1016/j.compbiomed.2024.108206 38430745

[27] Want EJ, O’Maille G, Smith CA, Brandon TR, Uritboonthai W, Qin C, et al. Solvent-dependent metabolite distribution, clustering, and protein extraction for serum profiling with mass spectrometry. Anal Chem. 2006;78(3):743–52. doi: 10.1021/ac051312t 16448047

[28] Safarchi A, Al-Qadami G, Tran CD, Conlon M. Understanding dysbiosis and resilience in the human gut microbiome: biomarkers, interventions, and challenges. Front Microbiol. 2025;16:1559521. doi: 10.3389/fmicb.2025.1559521 40104586 PMC11913848

[29] Da Silva Morais E, Grimaud GM, Warda A, Stanton C, Ross P. Genome plasticity shapes the ecology and evolution of *Phocaeicola dorei* and *Phocaeicola vulgatus*. Sci Rep. 2024;14(1):10109. doi: 10.1038/s41598-024-59148-7 38698002 PMC11066082

[30] Li Y, Zhang F, Zheng H, Kalasabail S, Hicks C, Fung KY, et al. Fecal DNA virome is associated with the development of colorectal neoplasia in a murine model of colorectal cancer. Pathogens. 2022;11(4):457. doi: 10.3390/pathogens11040457 35456132 PMC9025118

[31] Youssef RA, Sakr MM, Shebl RI, Saad BT, Aboshanab KM. Genomic characterization, in vitro, and preclinical evaluation of two microencapsulated lytic phages VB_ST_E15 and VB_ST_SPNIS2 against clinical multidrug-resistant Salmonella serovars. Ann Clin Microbiol Antimicrob. 2024;23(1):17. doi: 10.1186/s12941-024-00678-3 38360595 PMC10870556

[32] Oliveira RA, Ng KM, Correia MB, Cabral V, Shi H, Sonnenburg JL, et al. Klebsiella michiganensis transmission enhances resistance to Enterobacteriaceae gut invasion by nutrition competition. Nat Microbiol. 2020;5(4):630–41. doi: 10.1038/s41564-019-0658-4 31959968

[33] Nilsson H, Cardoso-Palacios C, Haggård-Ljungquist E, Nilsson AS. Phylogenetic structure and evolution of regulatory genes and integrases of P2-like phages. Bacteriophage. 2011;1(4):207–18. doi: 10.4161/bact.1.4.18470 23050214 PMC3448106

[34] Wegorzewska MM, Glowacki RWP, Hsieh SA, Donermeyer DL, Hickey CA, Horvath SC, et al. Diet modulates colonic T cell responses by regulating the expression of a Bacteroides thetaiotaomicron antigen. Sci Immunol. 2019;4(32):eaau9079. doi: 10.1126/sciimmunol.aau9079 30737355 PMC6550999

[35] Kim DS, Park Y, Choi J-W, Park S-H, Cho M-L, Kwok S-K. Lactobacillus acidophilus supplementation exerts a synergistic effect on tacrolimus efficacy by modulating Th17/Treg balance in lupus-prone mice via the SIGNR3 pathway. Front Immunol. 2021;12:696074. doi: 10.3389/fimmu.2021.696074 34956169 PMC8704231

[36] Zhang Z, Liu L, Tang H, Jiao W, Zeng S, Xu Y, et al. Immunosuppressive effect of the gut microbiome altered by high-dose tacrolimus in mice. Am J Transplant. 2018;18(7):1646–56. doi: 10.1111/ajt.14661 29316256

[37] Jin S, Xu H, Yang C, O K. Regulation of oxidative stress in the intestine of piglets after enterotoxigenic Escherichia coli (ETEC) infection. Biochim Biophys Acta Mol Cell Res. 2024;1871(5):119711. doi: 10.1016/j.bbamcr.2024.119711 38574824

[38] Swarte JC, Douwes RM, Hu S, Vich Vila A, Eisenga MF, van Londen M, et al. Characteristics and dysbiosis of the gut microbiome in renal transplant recipients. J Clin Med. 2020;9(2):386. doi: 10.3390/jcm9020386 32024079 PMC7074359

[39] Jiang J-W, Ren Z-G, Lu H-F, Zhang H, Li A, Cui G-Y, et al. Optimal immunosuppressor induces stable gut microbiota after liver transplantation. World J Gastroenterol. 2018;24(34):3871–83. doi: 10.3748/wjg.v24.i34.3871 30228781 PMC6141331

[40] Jennings DL, Bohn B, Zuver A, Onat D, Gaine M, Royzman E, et al. Gut microbial diversity, inflammation, and oxidative stress are associated with tacrolimus dosing requirements early after heart transplantation. PLoS One. 2020;15(5):e0233646. doi: 10.1371/journal.pone.0233646 32469966 PMC7259664

[41] Wang K, Dong H, Qi Y, Pei Z, Yi S, Yang X, et al. Lactobacillus casei regulates differentiation of Th17/Treg cells to reduce intestinal inflammation in mice. Can J Vet Res. 2017;81(2):122–8. 28408780 PMC5370538

[42] Tourret J, Willing BP, Dion S, MacPherson J, Denamur E, Finlay BB. Immunosuppressive treatment alters secretion of ileal antimicrobial peptides and gut microbiota, and favors subsequent colonization by uropathogenic Escherichia coli. Transplantation. 2017;101(1):74–82. doi: 10.1097/TP.0000000000001492 27681266

[43] Ren Z, Jiang J, Lu H, Chen X, He Y, Zhang H, et al. Intestinal microbial variation may predict early acute rejection after liver transplantation in rats. Transplantation. 2014;98(8):844–52. doi: 10.1097/tp.0000000000000334 25321166 PMC4206351

[44] Zhang X, Shen D, Fang Z, Jie Z, Qiu X, Zhang C, et al. Human gut microbiota changes reveal the progression of glucose intolerance. PLoS One. 2013;8(8):e71108. doi: 10.1371/journal.pone.0071108 24013136 PMC3754967

[45] Chandok N, Watt KD. Burden of de novo malignancy in the liver transplant recipient. Liver Transpl. 2012;18(11):1277–89. doi: 10.1002/lt.23531 22887956

[46] Gopalakrishnan V, Helmink BA, Spencer CN, Reuben A, Wargo JA. The influence of the gut microbiome on cancer, immunity, and cancer immunotherapy. Cancer Cell. 2018;33(4):570–80. doi: 10.1016/j.ccell.2018.03.015 29634945 PMC6529202

[47] Moini M, Schilsky ML, Tichy EM. Review on immunosuppression in liver transplantation. World J Hepatol. 2015;7(10):1355–68. doi: 10.4254/wjh.v7.i10.1355 26052381 PMC4450199

[48] Ponziani FR, Nicoletti A, Gasbarrini A, Pompili M. Diagnostic and therapeutic potential of the gut microbiota in patients with early hepatocellular carcinoma. Ther Adv Med Oncol. 2019;11. doi: 10.1177/1758835919848184 31205505 PMC6535703

[49] Jorgenson MR, Descourouez JL, Siodlak M, Tjugum S, Rice JP, Fernandez LA. Efficacy and safety of probiotics and synbiotics in liver transplantation. Pharmacotherapy. 2018;38(7):758–68. doi: 10.1002/phar.2130 29804307

[50] Shasthry SM. Fecal microbiota transplantation in alcohol related liver diseases. Clin Mol Hepatol. 2020;26(3):294–301. doi: 10.3350/cmh.2020.0057 32570299 PMC7364360

[51] Yu L, Zhang C, Zhai Q. Gut microbes participate in host polyamine metabolism. Proc Natl Acad Sci U S A. 2024;121(45):e2419368121. doi: 10.1073/pnas.2419368121 39467143 PMC11551406

[52] Martín R, Rios-Covian D, Huillet E, Auger S, Khazaal S, Bermúdez-Humarán LG, et al. Faecalibacterium: a bacterial genus with promising human health applications. FEMS Microbiol Rev. 2023;47(4):fuad039. doi: 10.1093/femsre/fuad039 37451743 PMC10410495

